# [Retracted] A disintegrin and metallproteinase 15 knockout decreases migration of fibroblast‑like synoviocytes and inflammation in rheumatoid arthritis

**DOI:** 10.3892/mmr.2024.13165

**Published:** 2024-01-17

**Authors:** Jinliang Gao, Wei Zheng, Liming Wang, Bailin Song

Mol Med Rep 11: 4389–4396, 2015; DOI: 10.3892/mmr.2015.3302

Following the publication of this paper, it was drawn to the Editors’ attention by a concerned reader that certain of the western blotting assay data shown in Figs. 1B and 5A and the histological data shown in Fig. 6C were strikingly similar to data appearing in different form in other articles written by different authors at different research institutes that had either already been published elsewhere prior to the submission of this paper to *Molecular Medicine Reports*, or were under consideration for publication at around the same time. In view of the fact that certain of these data had already apparently been published previously, the Editor of *Molecular Medicine Reports* has decided that this paper should be retracted from the Journal. The authors were asked for an explanation to account for these concerns, but the Editorial Office did not receive a reply. The Editor apologizes to the readership for any inconvenience caused.

